# Exploiting Unreduced Gametes for Improving Ornamental Plants

**DOI:** 10.3389/fpls.2022.883470

**Published:** 2022-06-06

**Authors:** Li Xie, Li-zhen Ke, Xiao-qi Lu, Jianjun Chen, Zhi-sheng Zhang

**Affiliations:** ^1^College of Forestry and Landscape Architecture, South China Agricultural University, Guangzhou, China; ^2^Mid-Florida Research and Education Center, Environmental Horticulture Department, Institute of Food and Agricultural Sciences, University of Florida, Apopka, FL, United States

**Keywords:** unreduced gametes, sexual polyploidization, plant breeding, ornamental plants, polyploid cultivars

## Abstract

The formation of gametes with somatic chromosome number or unreduced gametes (2*n* gametes) is an important process involved in the origin of polyploid plants in nature. Unreduced gametes are the result of meiotic mutations occurring during micro- and mega-sporogenesis. 2*n* gametes have been identified or artificially induced in a large number of plant species. Breeding of plants through 2*n* gametes can be advantageous because it combines genetic effects of polyploidy with meiotic recombination and sexual hybridization to produce tremendous genetic variation and heterosis. 2*n* gametes also occur in ornamental plants, but the potential of using 2*n* gametes in ornamental plant breeding has not been extensively exploited. Ornamental plants are primarily produced for their esthetic appearance and novelty, not for food and yield, and they can be readily propagated through vegetative means. Triploids, tetraploids, and plants with even higher ploidy levels produced through 2*n* gametes can be propagated through tissue culture to fix their phenotypes, thus leading to the development of new cultivars. In this review article, we intend to discuss the mechanisms underlying the formation of 2*n* gametes, techniques for 2*n* gamete identification, methods for enhancing 2*n* gamete formation, and the current status in the use of 2*n* gametes for development of novel ornamental plants. We believe that polyploidy breeding through 2*n* gametes represents a viable way of developing new cultivars, new species, and even new genera of ornamental plants.

## Introduction

Unreduced gametes are referred to as male or female gametes that have somatic chromosome numbers, thus they are also known as 2*n* gametes. This phenomenon as an important evolutionary force, however, was not recognized until the early last century. Prior to the 1920s, it was widely accepted that polyploidy in plants resulted from hybridization followed by chromosome doubling (Winge, 1917); Karpechenko (1927) was probably among the first to notice the occurrence of unreduced gametes. He believed that tetraploid *Oenothera*, *Primula*, *Solanum*, and *Datura* were not from the result of hybridization followed by chromosome doubling. In his study of hybridization between *Raphanus* and *Brassica*, Karpechenko (1927) found that hexaploid plants were not derived from doubling of triploid zygotes but caused by unreduced gametes from meiotic failure. The formation of unreduced gametes was further explained by Buxton and Darlington (1931) on amphiploid *Digitalis*, and they stated that the omission of reduction during the meiosis was the cause of somatic chromosome numbers. Subsequently, unreduced gametes were documented in an increasing number of plant species. Harlan and DeWet (1975) reported that unreduced gametes occurred in 85 plant genera. Recent studies showed that 2*n* gametes occur in a wide range of plants.

Unreduced gametes occur not only in plants but also in green algae, insects, chickens, mammals, birds, fish, and amphibians and have been considered a primary mechanism for polyploid formation (Mason and Pires, 2015). In plants, polyploids are not blind alleys or evolutionary dead-ends as claimed by Mayrose et al. (2011). Unreduced gametes facilitate polyploid formation and interploidy gene flow in mixed ploidy populations, resulting in increased genetic variation, fitness, heterozygosity, and breeding success. Additionally, 2*n* gamete formation is an essential component of apomixis Ravi et al., 2008 and an important way for the restoration of *F*_1_ hybrid fertility (De Storme and Geelen, 2013). Furthermore, 2*n* gamete formation generates novel genetic and genomic variation including synthesizing polyploid species, promoting plants to explore new environmental niches, and outcompeting their diploid progenitors.

This article is intended to review the occurrence of 2*n* gamete in ornamental plants, mechanisms underlying 2*n* gamete formation, the identification and use of 2*n* gametes for development of new ornamental cultivars. Ornamental plants are those grown for decoration and beautification of indoor and outdoor environments, not for food; thus, they are valued for their esthetic appearance, not for their yield. A large number of plants are produced as ornamental plants, including floriculture crops, ornamental shrubs, trees, grasses, and bamboos as well as ornamental aquatic plants (Chen, 2021). A significant number of ornamental plants are propagated vegetatively through natural means, such as bulbs, corms, runners, or artificial means like cutting, grafting, layering, or tissue culture. Evidence shows that 2*n* gametes have played a fundamental role in the development of polyploid cultivars, species, and even genera of ornamental plants, demonstrating the viability of sexual polyploidization in plant evolution and speciation.

## Mechanisms of 2*n* Gamete Formation

**FIGURE 1:**
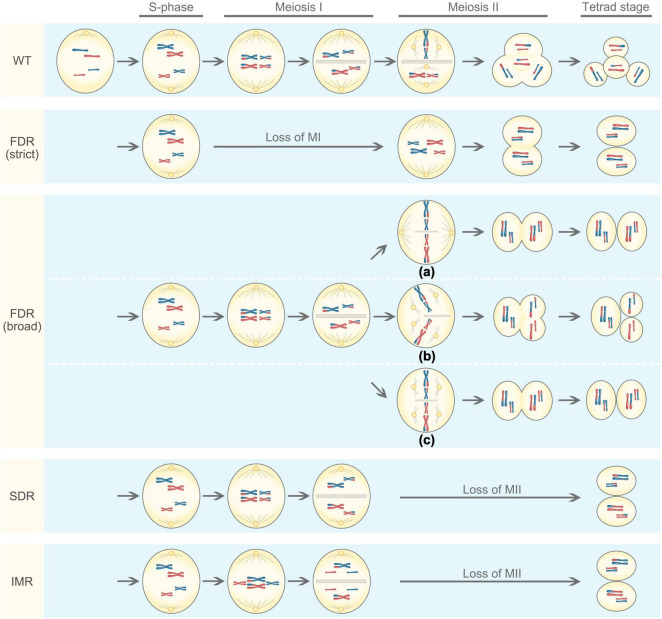
Mechanisms underlying the formation of 2*n* gametes based on the genotypic outcome. For simplicity, the meiotic cell is diploid and only contains two chromosomes that are fully heterozygous (blue and red chromosomes obtained from genetically different parents). WT represents normal meiosis, resulting in four haploid cells. There are two types of first division restitution (FDR): (1) In strict FDR, there was no chromosome pairing and recombination, and chromosomes directly advance to the second division, resulting in 2*n* gametes that are genetically identical to the parent. (2) In broad FDR, meiosis I is not lost, chromosomes pair and undergo recombination, but the orientation and position of the spindles in meiosis II are disturbed, often being parallel (a), tripolar (b), or fused (c). As a result, the diploid cell is predicted to contain non-sister chromosome (d’Erfurth et al., 2008). In second division restitution (SDR), meiosis I occurs normally with homologous chromosomes pairing and recombination, they divide reductionally followed by cytokinesis to produce a dyad. In meiosis II, however, the centromeres of the half-bivalents divide, but the chromatids do not migrate to the poles, resulting two 2*n* gametes. Indeterminant meiotic restitution (IMR) shows characteristics similar to FDR and SDR. During the first meiotic division, some bivalents disjoin reductionally as in SDR, while some univalents divide equationally as in FDR.

Unreduced gametes generally arise from meiotic defects. Meiosis is a process of producing haploid cells during which diploid cells undergo DNA replication, followed by two rounds of cell divisions known as meiosis I and meiosis II (Figure 1). In meiosis I, homologous chromosomes pair with each other and undergo genetic recombination, a process allowing to exchange genetic information through crossover. The homologous chromosomes are then separated, resulting in two haploid cells having half the number of chromosomes as the parental cell, thus meiosis I is a reductional division. Meiosis II resembles mitosis where sister chromatids are separated from each other, producing four cells with reduced chromosome number, this process is known as an equational division. However, meiotic defects can occur, including the omission of the first or second meiotic division, abnormal spindle morphology in the second division, or disturbed cytokinesis (Bretagnolle and Thompson, 1995; Ramanna and Jacobsen, 2003). Meiosis restitution is the predominant mechanism of 2*n* gamete formation in plants (Brownfield and Kohler, 2011; De Storme and Geelen, 2013). There are three main mechanisms underlying the formation of 2*n* gametes in plants: first division restitution (FDR), second division restitution (SDR), and indeterminant meiotic restitution (IMR).

### The First Division Restitution

In FDR, pairing and split-up of homologous chromosomes fail to occur or occur at a low frequency in meiosis I (Tang and Luo, 2002); but the second division proceeds normally, resulting in two sister chromatids of homologous chromosomes to move to opposite poles (Hermsen, 1984). There are two types of FDR: a strict sense and a broad sense (Sun et al., 2021). In the strict sense, there was no pairing and recombination, and chromosomes directly advance to the second division, resulting in 2*n* gametes that are genetically identical to the parent. Such an FDR fully maintains parental heterozygosity and epistasis. In the broad sense, meiosis I is not lost, chromosomes pair and undergo recombination, but the orientation and position of the spindles in meiosis II are disturbed (d’Erfurth et al., 2008), often being parallel (a), tripolar (b), or fused (c; Figure 1). As a result, the broad-sense FDR produces either two 2*n* gametes or one 2*n* gametes with two haploid gametes. In this case, 2*n* gametes partially retain the parental heterozygosity. However, the occurrence of crossover may increase genetic variation and also allow the introgression of genes of interest in breeding.

### The Second Division Restitution

In contrast to FDR, the first meiotic division occurs normally in SDR. Homologous chromosomes pair with recombination, and they divide reductionally followed by cytokinesis to produce a dyad. In meiosis II, however, the centromeres of the half-bivalents divide, but the chromatids do not migrate to the poles (Figure 1). The resulting 2*n* gametes are homozygous from the centromere to the first crossover but maintain parental heterozygosity at the telomeric side (Ramanna and Jacobsen, 2003). As a result, 2*n* gametes derived from SDR have reduced heterozygosity and show a substantial loss of parental epistasis (Peloquin et al., 2008). In general, SDR is rare in hybrids because all chromosomes are not appropriately paired as bivalents, and it tends to occur only in hybrids with closely related genomes (Ramanna and Jacobsen, 2003). The presence of cytokinesis and the formation of a cell wall after the telophase I is characteristic for most of the monocot plants (Bielig et al., 2003; Boldrini et al., 2006).

### Indeterminant Meiotic Restitution

The occurrence of IMR was first reported in interspecific hybrids of ornamental lily (Longiflorum × Asiatic lily; Lim et al., 2001). IMR shows characteristics similar to FDR and SDR in which both univalents and bivalents are formed at metaphase I. During the first meiotic division, some bivalents disjoin reductionally as in SDR, while some univalents divide equationally as in FDR (Figure 1), which give rise to 2*n* gametes with an odd number of parental chromosomes. Unreduced gametes produced in IMR only partially retain parental heterozygosity at the centromere (Zhou et al., 2008).

### Origin of 2*n* Gametes in Relation to Ornamental Plant Breeding

The origin of 2*n* gametes has profound effects on breeding of ornamental plants. In general, 2*n* gametes derived from FDR are more advantageous than those from SDR for transferring parental heterozygosity (Barcaccia et al., 2003; Zhang et al., 2009; Dewitte et al., 2012). In potato, FDR is known to be more than twice as effective as SDR in transmitting parental heterozygosity (Barone et al., 1995; Peloquin et al., 2008). Furthermore, progenies bred by FDR 2*n* gametes have more vigorous growth due to the higher allelic diversity (Yao et al., 2013). FDR is the basic mechanism of 2*n* pollen formation in *Alstroemeria* (Ramanna et al., 2003), *Begonia* (Dewitte et al., 2010a), *Lilium* (Lim et al., 2001; Barba-Gonzalez et al., 2008; Zhou et al., 2008; Khan et al., 2010), and *Tulipa* (Marasek-Ciolakowska et al., 2014). This is probably attributed to the chromosomal composition of FDR gametes that are more balanced and more viable than those from SDR and IMR. Additionally, 2*n* gametes derived from FDR or IMR with crossovers can increase genetic variation in polyploid progenies as well as the extent of introgressions Barba-Gonzalez et al., 2005. Genomic *in situ* hybridization (GISH) analysis confirmed the presence of recombinant chromosomes in FDR derived 2*n* gametes in meiotic polyploids in *Lilium* (Khan et al., 2009a,b; Xie et al., 2010) and *Tulipa* (Marasek-Ciolakowska et al., 2012, 2014).

## Methods for Identification of 2*n* Gametes

The identification of 2*n* gametes is largely focused on pollen as it is more convenient to isolate than egg cells. Common methods include pollen size measurements, flow cytometric detection of pollen DNA content, analysis of the microsporogenesis, and ploidy analysis of the progeny (Loginova and Silkova, 2017; Hwang et al., 2020). The identification of 2*n* eggs is complicated, which is performed by cytological examination using paraffin section, along with the ploidy analysis of the progeny.

### Pollen Size

The traditional approach for identifying 2*n* pollen is based on pollen morphology. 2*n* pollen is commonly known as giant pollen (Bretagnolle and Thompson, 1995), which is defined as a pollen with a diameter greater than 1.5 times that of the normal one. This criterion is based on the assumption that the doubling of DNA content in 2*n* gametes would approximately double the pollen cell volume (Mason et al., 2011). In ornamental plants of *Agave* (Gomez-Rodriguez et al., 2012), *Begonia* (Dewitte et al., 2009), *Dianthus* (Zhou et al., 2015), *Hibiscus* (van Laere et al., 2009), and *Rosa* (Crespel et al., 2006), the diameter of the 2*n* pollen is about 30% larger than that of the haploid pollen. The presence of large 2*n* pollen grains results in a bimodal distribution of pollen sizes instead of a normal distribution (De Storme and Geelen, 2011). Although the size distribution between the pollen grains sometimes overlaps, a threshold value of the pollen grain size is often used to select individuals that produce 2*n* gametes (Sugiura et al., 2000; Crespel et al., 2006). However, caution should be given when using this method to guide 2*n* gamete identification because giant pollen does not necessarily prove doubled DNA content. Another disadvantage of this screening technique is the broad overlap in size distribution between small and large pollen in some genera, such as grasses. In these cases, the frequency of 2*n* pollen based on size is difficult to determine. Thus, other methods should be used to confirm the association between giant pollen and 2*n* pollen, and supplementary evaluation of pollen viability is necessary for breeding purposes.

### Flow Cytometry

Flow cytometry has been widely used to measure pollen nuclear DNA content in order to understand pollen development and detect the presence of 2*n* pollen (Bino et al., 1990; Dewitte et al., 2009; van Laere et al., 2009; Zhu et al., 2014; Zhang et al., 2021). Estimating male 2*n* gametes with flow cytometry entails extracting nuclei from a large number of pollen grains, staining them with a DNA-selective fluorochrome, and generating fluorescence histograms with peaks corresponding to groups of nuclei with different DNA content. Flow cytometric analysis compares the DNA content of pollen nuclei to the DNA content of somatic leaf tissue. Pollen nuclei are expected to have only half of the DNA content (1C) compared to nuclei from somatic cells (2C) of the same plant. Consequently, 2*n* pollen have a nuclear DNA content equal to that somatic cells.

### Cytological Observation

The occurrence in 2*n* pollen is associated with the presence of monads, dyads, or triads during microsporogenesis (Negri et al., 1995; Fernandez et al., 2010; Chung et al., 2013; Nakato and Masuyama, 2021; Xu et al., 2021; Zhang et al., 2021) except 2*n* gamete formation that is the result of pre- or post-meiotic restitution. Analysis of microsporogenesis may therefore provide an alternative method to identify 2*n* pollen, but this method does not provide any information about pollen viability. Cytological staining is carried out with dyes, such as acetocarmine, aceto-orcein, or fuscin, resulting in the visualization of chromatins. With the advent of a new cytological methods, immunostaining, in combination with the use of propidium iodide (PI) and 4’,6’-diamidino-2-phenylindol and spindle (anti-α-tubulin antibodies), cellular components involved in the division can be more accurately determined. Recently, immunostaining using antibodies to phospho-histone H3 (Ser10), which is characterized by localization along the entire chromosome length in the first meiotic division and only in the centromeric region in the second division, has made it possible to distinguish the stages of meiotic division (Loginova and Silkova, 2016).

### Analysis of Progenies

Ploidy analysis of progenies (usually using flow cytometry) can reveal the presence of 2*n* gametes in parent plants. This method has been used frequently in ornamental plant breeding. In breeding of *Hibiscus*, five *F*_2_ hexaploid plants were isolated from self-pollinated hybrids of tetraploid *F*_1_, which was developed from the cross between *H. syriacus* “Oiseau Bleu” (4*x*) and *H. paramutabilis* (4*x*). The occurrence of hexaploidy indicated that the *F*_1_ hybrids must produce unreduced eggs since no unreduced pollen could be detected in the *F*_1_ hybrids (van Laere et al., 2009); Zhou et al. (2017) identified seven tetraploid hybrid plants from 12 progenies obtained from five crossing combinations between a tetraploid *Dianthus caryophyllus* “Butterfly” and diploid cultivars, suggesting that 2*n* male gametes were involved in polyploid formation. In lily breeding, four odd-allotetraploid seedlings were obtained from an interploidy cross, *Lilium* LA × AAAA. This result implied that the intergenomic variation was caused by 2*n* eggs, which was confirmed by GISH (Xiao et al., 2021). Progeny analysis, however, is time consuming with no guarantee of information about the production frequency of 2*n* gametes in the parental plants (Bretagnolle and Thompson, 1995) due to the differences in pollen viability, germination speed, or pollen tube growth between haploid and 2*n* pollen.

### Genomic *in situ* Hybridization

The use of molecular cytological techniques, such as GISH and fluorescent *in situ* hybridization in combination with marker analysis, such as amplified fragment length polymorphism on meiocytes or polyploid progeny provides more accurate or additional information on the mechanisms behind 2*n* gamete formation (Barba-Gonzalez et al., 2005; Chung et al., 2013; Zhang et al., 2021). Molecular cytological approaches have been successfully used in the case of allopolyploids, where the constituent genomes can be clearly discriminated (Xi et al., 2015). Through DNA *in situ* hybridization, genomes of allopolyploids can be more critically assigned and intergenomic translocations and recombination can be detected, which has been used in Gasteria-Aloe hybrids (Takahashi et al., 1997), *Alstroemeria* species (Ramanna et al., 2003), and *Lilium* species (Karlov et al., 1999; Lim et al., 2001; Barba-Gonzalez et al., 2005). As such, GISH can also be used to discover the mechanism of 2*n* gamete formation (Karlov et al., 1999).

## Occurrence of 2*n* Gametes in Ornamental Plants

Unreduced gametes can occur naturally *via* the mechanisms described above and artificially through the manipulation of environmental conditions or use of specific chemicals. Thus, the natural occurrence is the formation of 2*n* gametes spontaneously without artificial intervention. Up to now, naturally occurring unreduced gametes have been reported in more than 40 genera across 60 species and hybrid progenies of ornamental plants (Table 1), and artificially induced 2*n* gamete formation has been reported in at least 10 genera of ornamental plants (Table 2).

**TABLE 1:** Natural occurrence of 2*n* gametes in ornamental plants.

Plant	Type of unreduced gametes^z^	Identification method	Frequency of 2*n* gamete occurrence (%)	References
*Achillea eriophora* (2*x*), *A. tenuifolia* (2*x*), *A. oxyodonta* (2*x*), *A. talagonica* (2*x*), *A. biebersteinii* (2*x*), *A. wilhelmsii* (4*x*), *A. vermicularis* (4*x*), *A. millefolium* (6*x*)	M	Pollen size	1.00–3.30 ^y^	Sheidai et al., 2009
*Adiantum pedatum*	–	Cytological observation	–	Rabe and Haufler, 1992
*Agave angustifolia* var. “Cimarrón,” *A. angustifolia* var. “Lineño,” *A. tequilana*	M	Pollen size and cytological observation	1.20–3.20	Gomez-Rodriguez et al., 2012
*Alstroemeria F*_2_ hybrids progenies	M	Cytological observation and GISH	0.21–0.59	Ramanna et al., 2003
*Aranda*	M	Cytological observation	Over 10%	Lee and Tham, 1988
*Begonia* “Anna Christina,” *B. dregei*, *B. pearcei, B. “*Bubbles,” *B. “*Spatflacier,” *B.* “Orococo,” *B.* “Florence Rita,” B276	M	Pollen size, cytological analysis, and flow cytometry	2–100 ^y^	Dewitte et al., 2009, 2010b
*Camellia* cultivar HJ (6*x*)	M	Pollen size, cytological analysis, flow cytometry, ploidy level of progenies	–	Zhang et al., 2021
*Centaurea pseudophrygian* (2*x*), *C. jacea* (4*x*)	M/F	Progeny analysis	–	Koutecky et al., 2011
*Cyclamen F*_1_ hybrid	M	Cytological analysis	–	Ishizaka, 1998
*Cymbidium sinense*, *C. lancifolium*, and 30 *F*_1_ hybrids of *C. sinense* × *C. lancifolium*	M	Cytological analysis	0.19, 0.22, 0–9.36	Guo et al., 2021
*C. sinense* “Qijianbaimo,” “Damo,” “Hezhihua,” “Xiaoxiang,” “Taipingyang,” *Cymbidium* hybrids “Dafeng,” “Yunv,” “45–17,” “45–32”	M	Cytological analysis and flow cytometry	0.15–4.03	Zeng et al., 2020
*Cyphoniandra betace* “B24,” “LA,” Variable type	–	Pollen size and cytological analysis	–	Pringle and Murray, 1992
*Datura stramonium* (4*x*)	M	Cytological observation	0.10 ^y^	Belling and Blakeslee, 1923
*Dactylis glomerat*a subsp. *castellala*, *D. lusitanica, D*. *aschersoniana*, *D. parthiana*, *D. himalayensis*, galician type, *D. juncinell*, *D. ibizensis*, *D. smithii*	M/F	Progeny analysis and cytological observation	0.14–14.35/ 0.10–25.68	Haan et al., 1992; Maceira et al., 1992
*Dendranthema grandiflora* (6*x*)	M	Flow cytometry	–	Bino et al., 1990
*Dianthus caryophyllus* “Promesa,” “Guernse Yellow,” “YunhongErhao,” “Red Barbara,” “L. P. Barbara,” “Nogalte,” “Arevalo”	M	Pollen size and cytological analysis	0–4.17	Zhou et al., 2015
*Diospyros* spp. (6*x*) staminate germplasm	M	Pollen size and ytological analysis	0–2.30	Xu et al., 2008
*Fuchsia Hatschbachii*, *F. boliviana*, *F. microphylla*, *F. encliandra*, *F. trumpetor, F. fulgens* × *F. magellanica, F. fulgens* × *F. splendens, F. triphylla* × *F. splendens*	M	Pollen size and cytological analysis	1–13	Talluri, 2011
*Geum* hybrids	M	Cytological analysis	86 ^x^	Gajewski, 1953
*Helianthus F*_1_ hybrids, 11 non-hybrids	M	Pollen size, cytological analysis, progeny analysis, and GISH	27.52 (hybrids), 0.56 (non-hybrid)	Liu et al., 2017
*Hibiscus F*_1_ hybrids (4*x*)	F, M	Pollen size, cytological analysis, flow cytometry, and progeny analysis	–	van Laere et al., 2009
*Hydrangea aspera F*_1_ hybrids (H1–10)	M	Pollen size, cytological analysis, and progeny analysis	–	Crespel and Morel, 2014
*Ipomoea coccinea*, *I. quamoclit*, *I. F*_1_ hybrids, *F*_2_ hybrids	M	Cytological analysis and progeny analysis	0.10–92.40	Eckenwalder and Brown, 1986
*Iris domestica*, *Iris dichotoma*, *F*_1_-5, *F*_2_-2, *F*_2_-3, BC_1_-S-2, BC_1_-S-3, BC_1_-S-4,BC_1_-Y −1, BC_1_-Y −2	M	Pollen size and cytological analysis	0.20–2.30	Xu et al., 2021
*Lantana camara* “Radiation” (4*x*), “UPL” (4*x*) “Gold” (4*x*), “Pink Caprice” (4*x*), “P604–1” (4*x*), “GDGHOP-36” (2*x*), “GDOP-4” (3*x*), “PCOP-6” (4*x*), “PKGHOP-1” (2*x*)	F	Progeny analysis	5.50–100	Czarnecki and Deng, 2009
*Lilium F*_1_ hybrids	M	Pollen size, cytological analysis, and flow cytometry	–	van Tuyl et al., 1989
*Lilium F*_1_ AuH hybrids	M	Cytological analysis, GISH, and progeny analysis	42	Chung et al., 2013
*Lilium F*_1_ hybrid “79418-2”	M	Cytological analysis	25.10	Lim et al., 2004
*Lilium F*_1_ LA hybrids	F	GISH and chromosome nomenclature	–	Xiao et al., 2021
*Lilium* LA hybrids “88542-24,” “88542-69,” “88542-52”	M	Cytological analysis, GISH, and FISH	3–30	Lim et al., 2001
*Lotus F*_1_ hybrid “1321/46”	M	Cytological analysis	1.55	Negri et al., 1995
*Lotus tenuis*	M	Cytological analysis and progeny analysis	–	Negri and Veronesi, 1989
*Madia citriodora* × *gracilis* (3*x*) *Layia pentachaeta* × *platyglossa* (2*x*) *Madia nutans* × *rammii* (2*x*)	M	–	37^x^ 14 ^x^ 11.60 ^x^	Clausen et al., 1945
*Microseris* hybrids	M	–	21 ^x^, 20 ^x^	Chambers, 1955
*Medicago coerulea*, *Medicago sativa*, *Medicago falcata*	F,M	Cytological analysis, and progeny analysis	–	Veronesi et al., 1986
*Mertensia echioides* P-1, P-2, P-3	M	Pollen size	4–8.50	Malik et al., 2014
*Pancratium maritimum*	M	Cytological observation	–	Konyar, 2017
*Papaver* hybrids	M	–	9.01 ^x^	Yasui, 1931
*Phalaenopsis* Timothy Christonpher (2*x*), *Dtps.* Mini Red Rose (2*x*), *P.* Little Mary (3*x*) *Dtps*. Taisuco Pixie (3*x*), *P*. Taisuco Yellow Ball (4*x*), *Dtps*. King Shang’s Beaut (4*x*)	M	Pollen size, cytological analysis, and flow cytometry	0.55–2.84	Zhu et al., 2014
*Phegopteris decursivepinnata* “3*x*-1” (3*x*), “3*x*-2” (3*x*)		Cytological analysis and progeny analysis	2.44, 3.18	Nakato and Masuyama, 2021
*Populus tomentosa* “B^111^,” *Populus tomentosa* × *Populus bolleana*	M	Pollen size and cytological analysis	14.3,51.2	Kang and Zhu, 1997
*Primula sieboldii* (2*x*, 3*x*, 4*x*) 53 cultivars	M	Pollen size	0–10.80 ^y^	Yamaguchi, 1980
*Primula* hybrids (3*x*)	F	Progeny analysis and flow cytometry	–	Hayashi et al., 2009
*Quamoclit* hybrids	M	–	2.41–6.90 ^x^	Kagawa and Nakajima, 1933
*Rosa hybrida* “H3”	M	Pollen size and progeny analysis	–	El Mokadem et al., 2002a
*Rosa hybrida* “H190,” “H95,” “H126,” “H61”	F	Progeny analysis	58,79,92,97	El Mokadem et al., 2002b
*Rose* hybrids “HW”	M	Pollen size and cytological analysis	0–9.60	Crespel et al., 2006
*Trifolium prutense* C_0_, C_1_, C_2_, C_3_	M	Pollen size and progeny analysis	0.04–47.38	Parrott and Smith, 1986
*Trifolium prutense* C_1_	F	Pollen size and progeny analysis	3.40	Parrott and Smith, 1986
*Trifolium prutense* “Arlington,” “Florex,” “Redman,” “C760”	F	Pollen size and progeny analysis	0.014–0.50	Parrott et al., 1985
*Turnera F*_1_ hybrids (5*x*)	M	Cytological analysis	0.03 2*n* or 4*n*	Fernandez et al., 2010; Kovalsky and Neffa, 2012

*^z^M and F represent male and female gametes, respectively.*

*^y^Indicates large pollen grains.*

*^x^Estimated by the relative frequency of dyads and tetrads during microsporogenesis.*

**TABLE 2:** Induction of 2*n* gametes in ornamental plants.

Induction method	Material	Type of unreduced gametes^z^	Identification method	Frequency of 2*n* gamete occurrence (%)	References
Caffeine	*Lilium F*_1_ Hybrids	M	Flow cytometry, GISH, and progeny analysis	NA ^y^	Lim et al., 2005
Colchicine	*Eucalyptus urophylla*	M	Pollen size and cytological analysis	1.33–28.71	Yang et al., 2016
	*Lilium* FA hybrids “Jiaoyang”	M	Pollen size and cytological analysis	33–83	Piao et al., 2020
	*Lilium* “Con. Amore,” “Acapulco”	F	Progeny analysis	1.20–9.50, 2.30–25.80	Wu et al., 2007
	*Lilium oriental* “Sorbonne”	M	Cytological analysis and flow cytometry	68	Liao et al., 2016b
	*Lilium* “Valdisole”	M	Flower bud sizes	1.50–3.20	Zang et al., 2010
	*Populus canescens*	M	Progeny analysis, flow cytometry, and cytological obervation	2.75–30.27	Zhou et al., 2020
	*Populus tomentosa* “B^111^” or *Populus tomentosa* × *Populus bolleana*	M	Pollen size and cytological analysis	38.70, 68.50–85.10	Kang and Zhu, 1997
	*Populus alba* × *Populus glandulosa*	F	Progeny analysis and cytological observation	NA	Li et al., 2008
	*Rosa* “Old Blush”	M	Pollen size and cytological analysis	0.99–15.83	Zhang et al., 2019a
	*Strelitzia reginae*	M	Pollen size and cytological analysis	20.30	Zheng et al., 2017
	*Zantedeschia* hybrid “Black magic,” “Flamingo”	F, M	Progeny analysis and cytological observation	NA	Li et al., 2011
Nitrous oxide	*Begonia. subvillosa, Begonia F*_1_ hybrids	M	Pollen size, progeny analysis, and flow cytometry	NA	Dewitte et al., 2010a
	*Lilium F*_1_ hybrids	F, M	Progeny analysis and cytological observation	NA	Barba-Gonzalez et al., 2006
	*Lilium* hybrids “Kitazawa-Wase,” “Raizan”	M	Pollen size and progeny analysis	NA	Sato et al., 2009
	Asiatic hybrid lilies “Mona,” “Alaska”	M	Pollen size, flow cytometry, and cytological observation	33–100,75–100	Akutsu et al., 2007
	*Lilium* hybrids	M	Pollen size, cytological analysis, and flow cytometry	NA	Nukui et al., 2011
	*Lilium* OT hybrids “Nymph,” “Gluhwein,” “Velloween”	M	Pollen size and cytological analysis	NA	Luo et al., 2016
	*Phalaenopsis amabilis*	M	Progeny analysis, flow cytometry, and cytological analysis	NA	Wongprichachan et al., 2013
	*Tulipa* “Ile de France,” “Transavia”	M	Pollen size and flow cytometry	17–85	Okazaki et al., 2005
Trifluralin	*Begonia cucullate*, *B. fischeri*, *B. subvillosa*	M	Pollen size, flow cytometry, and progeny analysis	NA	Dewitte et al., 2010a
	*Lilium* Hybrids	F	Progeny analysis, GISH, FISH, and glow cytometry	NA	Barba-Gonzalez et al., 2006
	*Lilium* LA hybrid “Bonsior”	M	Pollen size and cytological analysis	59.70	Feng et al., 2012
	*Rosa chinensis minima*	M	Pollen size and cytological analysis	NA	Zlesak et al., 2005
High temperature	*Rosa* hybrids “HW20,” “HW154,” “HW336”	M	Pollen size, cytological analysis, flow cytometry, and progeny analysis	0.10–0.80, 0.60–3.70, 0.70–4.70	Crespel et al., 2015
	*Rosa* hybrids “HW336”	M	Cytological analysis	1.10–24.50	Pecrix et al., 2011
	*Diospyros kak*i (6*x*)	M	Pollen size and cytological analysis	0.77–22.04	Mai et al., 2019
	*Populus pseudo-simonii*	M	Cytological analysis	20.78–63.09	Wang et al., 2017b
	*Populus adenopoda*	F	Progeny analysis, cytological observation, and flow cytometry	NA	Lu et al., 2013

*^z^M and F represent male and female gametes, respectively.*

*^y^NA, Not available from the publication.*

### Naturally Occurring 2*n* Gametes

Naturally occurring 2*n* gametes are mainly derived from two major sources: interspecific or intergeneric hybrids and odd-polyploids (Bretagnolle and Thompson, 1995). Interspecific and intergeneric hybrids have a greater chance to produce 2*n* gametes at a higher frequency than their parents (Ramsey and Schemske, 1998; Ramanna et al., 2003). The frequency of 2*n* male gamete formation in traditional cultivars of *Cymbidium* ranged from 0.5 to 1.0% but 2.5 to 4.03% in interspecific hybrids (Zeng et al., 2020). Unreduced gametes were produced in interspecific or intergeneric hybrids of *Alstroemeria* (Ramanna et al., 2003), *Cyclamen* (Ishizaka, 1998), *Cymbidium* (Zeng et al., 2020), *Hibiscus* (van Laere et al., 2009), *Impatiens* (Stephens, 1998), *Lilium* (Lim et al., 2001, 2004; Barba-Gonzalez et al., 2005), and red clover (*Trifolium*; Meredith et al., 1995; Table 1). Cytologically, interspecific hybrids either show no chromosome pairing or have abnormal pairing and the presence of lagging chromosomes, chromosome bridges, or univalent in meiosis (Trojak-Goluch and Berbec, 2003; Dewitte et al., 2012; Crespel and Morel, 2014; Wang et al., 2015). Two important features have been reported in 2*n* gamete formation among interspecific hybrids. First, 2*n* eggs and 2*n* pollen could be simultaneously produced by the same hybrid. Second, neither the two parents of the *F*_1_ hybrids nor their (*F*_2_) sexual polyploid progenies could produce 2*n* gametes in any notable frequencies (Ramanna and Jacobsen, 2003).

The second source for frequently producing 2*n* gametes is odd polyploids. Old polyploids are characterized by their karyotypic, genomic, and reproductive instability. As a result, their meiosis may produce gametes with different levels of ploidy, including 2*n* gametes. For example, triploid lily is generally sterile but can be used as a female parent to produce fertile progenies. In this case, there is an occurrence of 2*n* gametes. Thus, odd polyploids have been considered a source for 2*n* gamete formation, and triploids have been used as a bridge between diploids and tetraploids to produce higher polyploids (Köhler et al., 2010).

### Artificial Induction of 2*n* Gametes

Unreduced gametes can be induced by manipulation of environmental factors, temperature in particular and treatment with chemicals, such as nitrous oxide (N_2_O), trifluralin, colchicine, and oryzalin (Table 2). The treatments, depending on the magnitude, may cause meiosis abnormalities in microspore mother cell, including chromosome separation failure (chromosomal adhesion or backward chromosomes) and spindle abnormalities (parallel, fused, and tripolar spindles; Li et al., 2016; Liao et al., 2016b; Wang et al., 2017b). Of these, parallel and fused spindles, and premature cytokinesis result in the formation of dyads, and the tripolar spindles create the triads during the tetrad period (Wu et al., 2011). These abnormalities could lead to the occurrence of 2*n* pollen.

### Temperature Treatment

Either high or low temperatures have been shown to triggers 2*n* gamete production (Mason et al., 2011; Pecrix et al., 2011; De Storme et al., 2012; Crespel et al., 2015; Zhou et al., 2015; Li et al., 2016; Wang et al., 2017b; Mai et al., 2019); Lokker et al. (2005) grew four complete sterile lily genotypes in a phytotron with an extreme temperature fluctuation regime: four alternating periods of 10 and 30^°^C each day for 6 weeks and found that three of the four genotypes became fertile as evidenced by the production of viable 2*n* gametes. Pecrix et al. (2011) observed that the frequency of 2*n* pollen in *Rosa* plants after exposure to a high temperature gradient was up to 24.5% compared to the control treatment at 24^°^C. The 2*n* pollen mainly resulted from temperature-induced spindle mis-orientations in meiosis II.

Low-temperature treatment can also induce 2*n* gamete formation. A short period of cold stress at 4–5^°^C induced the production of diploid and polyploid pollens in *Arabidopsis* (De Storme et al., 2012). In *Datura* and *Achillea*, the frequency of 2*n* pollen formation was higher at low temperatures (Ramsey and Schemske, 1998; Ramsey, 2007); Mason et al. (2011) demonstrated that cold stress significantly stimulated 2*n* pollen production in *B. napus* × *B. carinata* interspecific hybrids. Zhang et al. (2019b) showed that low temperatures increased the frequency of SDR-type 2*n* female gametes in the diploid rubber clone GT1.

### Chemical Reagents Induction

Colchicine, oryzalin, trifluralin, N_2_O, and amiprophos-methyl have been commonly used for inducing polyploids and also 2*n* pollen formation (Younis et al., 2014). Colchicine has been used for inducing 2*n* pollen of *Begonia*, *Dianthus*, *Tulipa*, *Lilium*, and other ornamental plants (Okazaki et al., 2005; Akutsu et al., 2007; Wu et al., 2007; Sato et al., 2009; Dewitte et al., 2010a; Lai et al., 2015; Yang et al., 2016). N_2_O can inhibit microtubule polymerization, but not actin filament formation. It was reported that N_2_O effectively induced 2*n* gametes (both 2*n* pollen and 2*n* egg) in *Tulipa* (Okazaki et al., 2005), *Lilium* (Barba-Gonzalez et al., 2006; Akutsu et al., 2007), and *Begonia* (Dewitte et al., 2010a). N_2_O as a gas can readily penetrate tissue, thereby protecting the tissues from harmful aftereffects as soon as the gas is released (Ostergren, 1954; Kato and Geiger, 2002); Akutsu et al. (2007) showed that effects of N_2_O were optimal when treatments started during pollen mother cell progression to metaphase I. Using this technique, fertile 2*n* gametes were induced from sterile hybrids, but the efficiency of the treatment was genotype specific (Barba-Gonzalez et al., 2006; Dewitte et al., 2010b).

### Potential for Engineering 2*n* Gametes

With the advance in molecular biology, genes specifically responsible for 2*n* gamete formation have been increasingly identified. An *Arabidopsis* gene *DYAD/SWITCH1* (*SWI1*) was found to be responsible for the production of 2*n* female gametes, resulting in progenies with triploid plants. *AtPS1* (*Arabidopsis thaliana* parallel spindle 1) is another gene involved in 2*n* gamete formation in *Arabidopsis* (d’Erfurth et al., 2008). *AtPS1* mutants produce up to 65% of 2*n* pollens, pollination with the pollen resulted in a large number of triploid plants in the next generation. *ASMC5/6* (structural maintenance of chromosome 5/6) complex has been identified to be a crucial factor for preserving genome stability (Yang et al., 2021). *SMC5/6* mutants show an absence of chromosome segregation during the first and/or second meiotic division, producing 2*n* gametes. A comparison of the well-established meiotic mutants in alfalfa with the genes identified from *Arabidopsis* showed that nine proteins belonging to *A. thaliana* known for their involvement in 2*n* gamete production occurred in alfalfa, suggesting common lineage of genes implicated in 2*n* gamete formation (Palumbo et al., 2021). Molecular techniques, particular CRISPR/Cas9 could be used for engineering plants with increased production of either 2*n* pollen or 2*n* eggs and used for breeding of novel polyploid ornamental plants.

## Use of 2*n* Gametes for Improving Ornamental Plants

A large number of polyploid ornamental cultivars have been developed through the use of 2*n* gametes (sexual polyploidization). Table 3 lists some of those across 21 genera, which can be summarized as follows: (1) triploids developed from the cross of diploid × diploid in *Cymbidium, Hevea brasiliensis*, *Lilium*, *Phalaenopsis, Populus, Rosa*, *Vaccinium*, and *Zantedeschia*; (2) tetraploids derived from the cross of diploid × diploid, diploid × tetraploid, or tetraploid × diploid in *Alstroemeria*, *Calluna vulgaris*, *Chrysanthemum*, *Cymbidium, Dianthus*, *Lilium*, *Morus alba*, *Petunia hybrida*, *Phalaenopsis*, *Phegopteris*, *Rosa*, *Primula*, *Pyrus*, *Ranunculus cantoniensis*, *Trifolium pretense*, and *Zantedeschia*; (3) pentaploids produced from the cross of diploid × triploid, triploid × diploid, tetraploid × diploid, tetraploid × triploid, or diploid × hexaploid in *Fragaria*, *Lilium*, *Phalaenopsis*, *Rosa*, *Primula*, and *Phegopteris*; (4) hexaploids obtained from the cross of diploid × tetraploid, tetraploid × diploid, tetraploid × tetraploid, triploid × triploid, or diploid × octaploid in *Fragaria*, *Phalaenopsis*, *Primula*, and *Phegopteris*; and (5) octaploids selected from the cross of tetraploid × tetraploid in *Primula*. Additionally, sexual triploids of *Hevea* (Zheng et al., 1983), *Populus* (Zhang et al., 1992; Li et al., 1994; Kang et al., 2000; Guo et al., 2017), and *Zantedeschia* (Wu et al., 2011), as well as tetraploids of *Petunia* (Cai et al., 2020) and *Lilium* (Barba-Gonzalez et al., 2004) were also successfully developed using artificially induced 2*n* gametes.

**TABLE 3:** Polyploid cultivars of selected ornamental plants developed through the use of 2*n* gametes.

Species or genus	Polyploidy obtained	Parents	Changes in Characteristics	References
*Alstroemeria*	Tetraploid	*A. inodora* (2*x*) × *A. pelegrina* (4*x*)	NA ^z^	Ramanna et al., 2003
*Cymbidium*	Triploid	*C.* × “Dafeng” (2*x*) × *C. sinense* “Hezhihua” (2*x*)	Rounder flowers, wider sepal, petals, and lips	Zeng et al., 2020
	Triploid	*C.* × “Yunv” (2*x*) × *C. sinense* “Xiaoxiang” (2*x*)	More robust growth with rounder flowers	
	Triploid and tetraploid	*C.* × “Yunv” (2*x*) × *C. sinense* “Taipingyang” (2x)	NA	
	Triploid	*C.* × “45–32” (2*x*) × *C.* × “45–17” (2*x*)	NA	
	Tetraploid	*C.* × “45–32” (2x) × *C.* × “45–32” (2*x*)	NA	
*Chrysanthemum*	Tetraploid	*C. remotipinnum* (2*x*) × *C. chanetii* (4*x*)	Intermediate leaf size inherited from female while round-shape blade and petiole from male parents	Abd El-Twab and Kondo, 2007
*Cyclamen*	Tetraploid	*Cyclamen persicum* (2*x*) × *C. persicum* (4*x*) or reverse cross	Larger guard cells	Takamura and Miyajima, 1996
*Calluna*	Tetraploid	Hybrid cultivar 7705 of *C. vulgaris* (4*x*) × *C. vulgaris* (2*x*)	Semi-fertile triploids with reduced invasiveness	Przybyla et al., 2014
*Dianthus*	Amphidiploid and tetraploid	*D. isebsis* (2*x*) × *D. japonicas* (2*x*)	Increased growth vigor	Nimura et al., 2006
*Fragaria*	pentaploid	*F. vesca* (2*x*) × *F. elatior* (6*x*)	Vigorous plant growth with profusely flowers	Fedorova, 1934
	Hexaploid	Cultivar of *F. vesca L.*(2*x*) × cultivar of *F.* × *ananassa* Duch. (8*x*)	NA	Yanagi et al., 2010
*Hevea*	Triploid ^y^	GT1 (2*x*) × RRIC52 (2*x*)	Fast growth and resistant to albinism	Zheng et al., 1983
	Triploid	GT1 (2*x*) × seventeen rubber trees (2*x*)	NA	Yao et al., 2016
*Lilium*	Pentaploid	BC1 (3*x*) × LLAA (4*x*)	NA	Lim et al., 2003
	Tetraploid and hexaploid	4X-OA (4*x*) × OA hybrids of *Lilium* (2*x*)	NA	Barba-Gonzalez et al., 2004
	Triploid and tetraploid	OA hybrids of *Lilium* (2*x*) selfing progeny	NA	
	Triploid and tetraploid	Asiatic hybrids of *Lilium* or Oriental hybrids of *Lilium* (2*x*) × OA hybrids of *Lilium* (2*x*)	NA	
	Triploid	OA hybrids of *Lilium* (2*x*) × Asiatic hybrids of *Lilium* (2*x*)	NA	
	Tetraploid	F1 OA hybrids “Vivaldi” (2*x*) × F1 OA “951301-5” (4*x*)	NA	Barba-Gonzalez et al., 2006
	Triploid	F1 Longiflorum × Asiatic hybrids (2*x*) × Asiatic Lily cultivars (2*x*)	NA	Zhou et al., 2008
	Tetraploid	F1 Longiflorum × Asiatic hybrids (2*x*) × Asiatic Lily “Tresor” (4*x*)	Taller stems with robust growth, tolerance to diseases and heat, and increased occurrence in bulbil	Xiao et al., 2021
*Lantana*	Hexaploid	*L.*“Pink Caprice” (4*x*) selfing progeny or *L.*“Gold Caprice”(4*x*) selfing progeny	NA	Czarnecki and Deng, 2009
*Petunia*	Tetraploid ^y^	“Hongxia” (4*x*) × “Menghuan” (2*x*)	Organs become bigger	Cai et al., 2020
*Phalaenopsis*	Triploid and tetraploid	*P.* Timothy Christonphe (2*x*) × *P.* Timothy Christonphe (2*x*)	NA	Zhou et al., 2009
	Pentaploid	*P.* Timothy Christonphe (2*x*) × *P. aphrodite* (3*x*) Or *Phalaenopsis* (3*x*) × *Phalaenopsis* (2*x*)	NA	
	Tetraploid	*P.* Timothy Christonphe (2*x*) × *P.* Railin Red Angel (4*x*) Or *P.* Railin Red Angel (4*x*) × *P.* Timothy Christonphe (2*x*)	NA	
	Pentaploid	*P.* Brother Yellew Boy (4*x*) × *P.* M3004*9* (3*x*)	NA	
	Hexaploid	*P.* Ever Spring Light (4*x*) × *P.* HO’s French Fantasia (4*x*)	NA	
*Populus*	Triploid ^y^	(*P. alba* × *P. glandulosa*; 2*x*) × (*P. tomentosa* × *P. bolleana*; 2*x*) or reverse cross	Increased growth vigor	Kang et al., 2000
	Triploid	*P. pseudo-simonii* × *P. nig* “Zheyin3#” (2*x*) × *P. beijingensis* (2*x*)	Increased growth vigor	Liao et al., 2016a
	Triploid ^y^	*P. simonii* or *P. simonii* × *P. nigra var. Italica* (2x); *P. simonii* × (*P. pyramidalis* + *Salix matsudana*; 2*x*)	Increased cell size and growth vigor	Guo et al., 2017
	Triploid ^y^	(*P. tomentosa* × *P. bolleana*; 2*x*) × (*P. tomentosa* × *P. bolleana*; 2*x*)	Vigorous growth with greener leaves	Zhang et al., 1992
*Phegopteris*	Hexaploid	*P. decursivepinnata* (3*x*) selfing progeny	Robust increase of leaf size with vigorous growth of plants	Nakato and Masuyama, 2021
	Tetraploid and pentaploid	*P. decursivepinnata* (3*x*) × *P. decursivepinnata* (2*x*)	Tetraploid progeny had well-shaped leaves and increased growth vigor. Pentaploid progeny had small and irregularly indented leaves	
*Primula*	Triploid	*P. sieboldii* (2*x*) × *P. kisoana* (2*x*)	Flowers with a central eye that was pale-yellow or white, some had no eye at all	Kato and Mii, 2000
	Triploid	*P. sieboldii* (2*x*) × *P. obconica* (2*x*)	Leaves were abnormally folded	Kato et al., 2001
	Triploid and tetraploid	*P. malacoides* (2*x*) × *P. malacoides* (2*x*)	Compact growth style with round petals and larger flowers	Horn, 2004
	Tetraploid	*P. malacoides* (2*x*) × *P. malacoides* (4*x*)	Compact growth style with round petals and larger flowers	
	Tetraploid	*P. malacoides* (4*x*) × *P. malacoides* (2*x*)	Compact growth style with round petals and larger flowers	
	Tetraploid	*P. rosea* (2*x*) × *P. denticulata* (4*x*)	Increased stress tolerance	Hayashi et al., 2007
	Hexaploid and octaploid	*P. modesta* (4*x*) × *P. denticulata* (4*x*) or reverse cross	Vigorous growth with very high pollen fertility	Kato et al., 2008
	Tetraploid, pentaploid, and hexaploid	*P. denticulate* (4*x*) × *P. modesta* (2*x*)	NA	
	Tetraploid and hexaploid	*P. modesta* (2*x*) × *P. denticulata* (4*x*)	NA	
	Pentaploid	DDR hybrid (3*x*) × *P. denticulata* (4*x*)	Increased DNA content	Hayashi et al., 2009
*Pyrus*	Tetraploid	*P.* “Dayali” (4*x*) × *P.* “Pingguoli” (2*x*)	NA	Cao et al., 2002
*Ranunculus cantoniensis*	Tetraploid	*R. silerifolius* (2*x*) × *R. chinensis* (2*x*)	NA	Okada, 1984
*Rosa*	Triploid	*R. wichuraiana* (2*x*) × H3 (dihaploid)	NA	El Mokadem et al., 2002a,b
	Tetraploid	*R. hybrida* cv Anna (4*x*) × H3 (dihaploid)	NA	
	Tetraploid	*R. hybrida* var. F01473 (4*x*) × H3 (dihaploid)	NA	
	Tetraploid	*R. hybrida* var. FJV6 (4*x*) × H3 (dihaploid)	NA	
	Triploid and tetraploid	Several dihaploid plants of *Rosa* (2*x*) × H3 (dihaploid)	NA	
*Trifolium*	Triploid and tetraploid	*T.* cultivar “Arlington”-A (2*x*) × *T.* cultivar C51 (2*x*)	NA	Parrott et al., 1985
	Tetraploid	*T. pretense* (2*x*) × *T. pretense* (4*x*)	NA	
*Tulipa*	Triploid	*T. gesneriana* (2*x*) × *T. fosteriana* (2*x*)	Larger flower, sturdy stem and plant size	Marasek et al., 2006
	Triploid	*T. gesneriana* (2*x*) × F1 Darwin hybrid (2*x*)	NA	Marasek-Ciolakowska et al., 2014
	Tetraploid and pentaploid	*T. gesneriana* “Bolroy Silver”(3*x)* × F1 Darwin hybrid (2*x*)	NA	
*Zantedeschia*	Triploid ^y^	Pink persuasion (2*x*) × Black magic (2*x*) or Black persuasion (2*x*) × Pink magic (2*x*)	Increased leaf size with circular deformation	Wu et al., 2011

*^z^NA, Not available from the publication.*

*^y^Indicates the artificially induced 2n gametes.*

Polyploidization through 2*n* gametes represents a new trend in breeding of ornamental plants (Ramanna et al., 2012; Marasek-Ciolakowska et al., 2021). This is mainly due to the following factors: (1) Ornamental plants are prized by their novelty and esthetic appearance, including flower shape, size, and color; leaf shape, texture, color, and size; plant overall growth form and growth vigor (Henny and Chen, 2003). They are not cultivated for grain production; thus, there is little concern whether or not they are poor in seed production or sterile due to triploid block. Plants with unique phenotypes can be effectively propagated asexually using tissue culture technique to immediately increase the number of plants for commercial production. (2) An increasing number of ornamental plant species has been found to produce 2*n* gametes (Table 1). The frequency of 2*n* gamete occurrence typically ranges from 0.1 to 2.0%, but it could be much higher up to 10% in interspecific hybrids (Kreiner et al., 2017a,b; Sun et al., 2021). Many ornamental plants are actually interspecific hybrids (Kato and Mii, 2012). The higher frequencies offer a unique opportunity for breeders to manipulate chromosomes and develop new cultivars of ornamental plants (Table 3), which is described in the following subsections. (3) Polyploid plants, particularly those developed *via* 2*n* gametes generally have increased organ size, robust growth form, and improved tolerance to abiotic and biotic stresses. Sexual polyploidization using 2*n* gametes allows the introgression of desirable traits in interspecific breeding and results in genetic heterozygosity and heterosis (Barba-Gonzalez et al., 2004, 2005, 2006). Although polyploidization can be attained through mitotic chromosome doubling (Eng and Ho, 2019; Niazian and Nalousi, 2020), this approach does not result in introgression breeding due to the lack of intergenomic recombination. (4) The ornamental plant industry is a fast-growing sector in world agriculture (Chen, 2021). Ornamental plants represent the sixth largest agricultural commodity group in the United States European countries, such as Netherlands produces a large quantity of diverse floriculture crops. The ornamental plant industry in China is blooming. The total turnover for floriculture crops (excluding ornamental trees and shrubs and ornamental grasses and bamboo) was estimated to be $300 billion (Azadi et al., 2016). A key driving force for the continuous growth of the ornamental plant industry is the demand for new cultivars with novel esthetic value (Henny and Chen, 2003; Noman et al., 2017). Thus, polyploidization through 2*n* gametes has been increasingly used for improving ornamental plants, and this is particularly true in bulbous and orchid crops. Most single and double flowered cultivars of *Hippeastrum* on the market are tetraploid. The majority of modern intersectional cultivars of *Lilium* are triploids, and some commercial ones are aneuploids. In the genus *Narcissus*, nearly 75% of cultivars are tetraploid, but only 12% each for diploid and triploid cultivars. Many tulip, chrysanthemums, and cultivated orchids are polyploid. Most modern commercially valuable rose cultivars are tetraploids. In fact, the availability of these neopolyploids is largely attributed to the functionality of 2*n* gametes used in breeding, which are briefly discussed as follows:

### Cultivar Development Through Interploidy Crosses

Developing new cultivars through interploidy crosses is often difficult due to the difference in ploidy levels. However, the occurrence of 2*n* gametes can greatly facilitate interploidy crosses, resulting in the development of new polyploid cultivars. The interploidy crosses include 2*x* × 4*x*, 4*x* × 2*x*, or 2*x* × 3*x.* Hybridization of tetraploids with diploids or vice versa produced triploid semperflorens *Begonia* and *Begonia rex* (Horn, 2004; Marasek-Ciolakowska et al., 2016) as well as triploids *Lilium* (Lim et al., 2003; Zhou et al., 2008) and *Tulipa* (Kroon and Van Eijk, 1977; van Scheepen, 1996). Triploid *Aloineae* (Brandham, 1982), *Dactylis* (Jones and Borrill, 1962), *Lilium* (Lim et al., 2003), *Primula* (Hayashi et al., 2009), and *Tulipa* (Marasek-Ciolakowska et al., 2014) crossed with tetraploid counterparts resulted in pentaploid plants, respectively. *Hydrangea macrophylla* is one of the most economically important ornamental crops worldwide, with United States sales of *Hydrangea* species topping $120 million in 2014. A recent study showed that diploid (2*n* = 2*x* = 36), triploid (2*n* = 3*x* = 54), tetraploid (2*n* = 4*x* = 72), and even aneuploid *H. macrophylla* are most fertile and produce viable offspring in interploidy crosses. Triploid and tetraploid offspring can be produced by hybridization of diploid with diploid individuals or by crossing diploid with tetraploid plants, and even crossing triploids with either diploid or tetraploid plants. Such interploidy crosses are due to production of unreduced gametes (Trankner et al., 2020). Triploid hydrangeas have thicker stems, large flowers, and larger stoma compared to full-sibling diploids. These findings explained the origin of triploid hydrangeas and also why there are more triploid cultivars are on the market than diploid cultivars (Alexander, 2020).

### Interspecific and Intergenic Cultivar Development

The availability of 2*n* gametes facilitates interspecific and intergenic hybrid development in ornamental plants. The aims of such hybridizations are to broaden genetic variability or transfer valuable traits, such as disease resistance and novel ornamental characteristics for developing new cultivars. *Begonia* is one of the largest genera of floriculture crops with more than 2,000 species. It has been divided into several groups based on the origin and growth characteristics. Among them, Elatior-hybrids represent about 88% of the total begonia production (Haegeman, 1979; Kroon, 1993). Most “Elatior” begonia were developed from crosses between different tuberous hybrid species (*B* × *tuberhydrida*) and *B. socotrana* (Marasek-Ciolakowska et al., 2016). Both spontaneous and induced 2*n* gametes have played important role in the interspecific hybrid development. For example, most “Elatior” hybrids are triploids, and a few are tetraploids (Marasek-Ciolakowska et al., 2016). The occurrence of 2*n* pollen was common in *Begonia* with a frequency varied from 1% in *Begonia* “Rubaiyat” to 100% in “Florence Rita” and B276 (Dewitte et al., 2009), of which FDR was the major mechanism underlying the 2*n* pollen formation (Dewitte et al., 2010a,b).

Tulip (*Tulipa* L.) is one the most popular bulbous crops, and its breeding has been aimed at the introgression of new flower colors and shapes, flower longevity, resistance to tulip breaking virus (TBV), *Botrytis tulipae*, and *Fusarium oxysporum* into commercial cultivars (Marasek-Ciolakowska et al., 2016). Interspecific crosses were made between *T. gesneriana* and *T. fosteriana* (TBV resistant) resulting in a series of cultivars including Darwin hybrids that are highly resistant to BVT virus (van Eijk et al., 1991; van Raamsdonk et al., 1995). More than 50 Darwin hybrid cultivars were developed (van Scheepen, 1996), which were largely derived from sexual polyploidization. This is because some Darwin triploids were fertile and could be backcrossed to *T. gesneriana*, and some F_1_ Darwin hybrids could produce 2*n* and haploid gametes, allowing the generation of polyploids (Marasek-Ciolakowska et al., 2016).

The occurrence of 2*n* gametes has also led to the formation of new species and new genera. Classical examples are tetraploid species of *Tragopogon mirus* and *T. miscellus* in the sunflower family (Soltis and Soltis, 2009). *T. mirus* (2*n* = 4*x* = 24) was derived from the cross of diploid *T. dubius* with diploid *T. porrifolius* (2*n* = 2*x* = 12), while *T. miscellus* (2*n* = 4*x* = 24) was developed from the cross of *T. dubius* with diploid *T. pratensis*. The underlying mechanism for the formation of the two species was explained by unreduced gametes produced by the diploid parents. An early example of synthesized genus is × *Aranda* orchids (Lee and Tham, 1988). This genus represents a group of intergenic hybrids developed from crosses between *Vanda* (2*n* = 2*x* = 38) and *Arachnis* (2*n* = 2*x* = 38). The initial hybrids (*F*_1_) of the two genera had 2*n* = 2*x* = 38 but were sterile. However, some of the hybrids produced 2*n* gametes at a rate up to 10%, and they were fertile as maternal parents. Backcross with either *Vanda* or *Arachnis* resulted in × *Aranda* hybrids with chromosome of 2*n* = 3*x* = 57. The vanda parents provide flower color and shape with the stacked strap leaves, and arachnis parents contributes curved, thin petals and fast growth characteristic. They have been widely used as cut flowers due to their vigorous growth and very abundant flowers. More than 200 such hybrids were developed prior to 1985.

### Allopolyploid Cultivar Development

There are two types of polyploidy: autopolyploids and allopolyploids. The former display polysomic inheritance, and the latter in most cases show disomic inheritance. In general, allopolyploid plants show higher heterozygosity and heterosis. Lily as one of the most important floriculture crops with four popular genomes: Asiatic (A genome), Longiflorum (L genome), Oriental (O genome), and Trumpet (T genome; van Tuyl and Arens, 2011). Using 2*n* gametes, along with cut style pollination and embryo rescue, LA, OA, LO, and OT hybrids were developed (Asano and Myodo, 1977; van Tuyl et al., 2000). By somatic chromosome doubling, allotetraploid hybrids of LALA, OAOA, LOLO, OTOT, and LTLT were produced (Xiao et al., 2021). As 2*n* gametes occurs in those *F*_1_ hybrids, interploidy crosses of 2*x* × 4*x* produced LAA, OTO, LOO, and AOA cultivars. Additionally, three odd-allotetraploid cultivars, namely Honesty (LAAA; Zhou et al., 2013; Xiao et al., 2019), Original Love (LAAA; Yang et al., 2019; Zheng, 2019), and Santa Rosa (LLLO; Zhang et al., 2012) were developed. Since LA can produce a small number of 2*n* egg, “Honesty” was developed from a cross of LA × AAAA. LALA can produce a large number of 2*n* gametes, “Original Love” was selected from the cross of LALA × AAAA. “Santa Rosa” was derived from the cross of LOLO × LLLL or vice versa. Most functional 2*n* gametes were formed through FDR, and a few were derived from IMR. Compared to diploid, triploid, and other tetraploid plants, the odd-allotetraploid cultivars have taller and stronger stems, produce more bulbils, and resist diseases (Xiao et al., 2021). As 2*n* gametes are largely produced through FDR, the heterosis is probably attributed to intergenomic differences in the hybrids. Allopolyploidy can confer additional advantages: novel genetic variation, and phenotypes different to the parent species can be produced through transgressive segregation and allelic heterosis.

### Triploid Cultivars Derived From 2*n* Gametes

Triploid plants can be recovered from the cross of 2*x* × 2*x*, 2*x* × 4*x*, 4*x* × 2*x*, or 2*x* × 3*x*, of which the cross of one parent that produces 2*n* gametes with another diploid parent is one of the most common practices (Wang et al., 2016). Studies showed that 2*n* gametes produced by a female parent plays important role in the successful formation of triploid plant (Ramsey and Schemske, 1998), and this is in part attributed to the appropriate endosperm balance number (Lu et al., 2013). Many triploid cultivars of *Narcissus* (Brandham, 1986), *Lilium* (Noda, 1986), *Crocus* (Ørgaard et al., 1995), and *Tulipa* (van Scheepen, 1996; Marasek et al., 2006) were developed from unreduced gametes of diploid parents. The early cultivars from the subgenus *Narcissus* were diploid, from which triploid cultivars arose in the latter half of 19th century due to the occurrence of 2*n* gametes. Subsequently, tetraploid *Narcissus* were developed by the end of the 19th century (Brandham, 1986). Tulips are important bulbous flowering plants with more than 8,000 cultivars in the market. Among them, Darwin hybrids represent an important group of cultivars grown for cut flowers, and they are triploids (2*n* = 3*x* = 36) developed from interspecific cross of *Tulipa gesneriana* (2*n* = 2*x* = 24) and *T. fosteriana* (2*n* = 2*x* = 24). GISH and median chromosome analyses showed that 24 chromosomes were derived from *T. gesneriana* and 12 chromosomes were from *T. fosteriana* (Marasek et al., 2006), suggesting that the one of the most popular groups of tulips, Darwin triploid hybrids were developed through 2*n* gametes derived from *T. gesneriana.*

A distinct characteristic of triploid plants is their sterility, known as triploid block, this is a phenomenon resulting in the formation of non-viable progeny after hybridization of plants with different ploidy. This is mainly due to the unbalanced meiotic chromosome segregation and endosperm imbalance (Köhler et al., 2010; Wang et al., 2017a). In ornamental plant breeding, triploid plants can be maintained through vegetative means, such as bulbous propagation in *Crocus, Lilium, Narcissus*, and *Tulipa* as well as micropropagation through tissue culture. Triploid ornamental plants generally have higher growth vigor, large flower size, sturdier stem, broader and thicker leaves, or more compact plants compared to their diploid progenitors because the energy that is normally devoted to seed production is used for the growth of flowers and other organs (Miyashita et al., 2009; Tiku et al., 2014). Furthermore, the sterility could be particularly useful for reducing the invasiveness of some ornamental plants. Some important ornamental plants are classified as invasive, as their seed production and dispersal by birds and other means could result in potential colonization of natural habitats that break the balance of native flora (Li et al., 2004). For example, *Lantana camara* is a popular ornamental plant but is considered an invasive species because its pollen can hybridize with an endangered relative *L. depressa* in Florida. Studies showed that triploid cultivars of *L. camara* had lowest pollen stainability at 9.3% compared to 64.6% in diploid and 45.1% in tetraploid cultivars (Czarnecki et al., 2014). Meanwhile, 2*n* female gametes were found to produce in diploid, triploid, and tetraploid cultivars (Czarnecki and Deng, 2009). Thus, the authors acknowledge that to develop triploid lantana, appropriate parental plants should be carefully selected (Czarnecki et al., 2012).

Triploid sterility, however, may not be completely correct. Increasing evidence shows that many triploids can be used as male or female parent in cross breeding programs (Lim et al., 2003; Zhou et al., 2008; Hayashi et al., 2009; Nakato and Masuyama, 2021). Pentaploids and hexaploids were produced by using of triploid as the parents in *Phalaenopsis* and *Primula*. In *Phalaenopsis*, no hybrids were produced from the cross of triploid × triploid; however, hexaploid was obtained from the self-pollinated progeny of triploid *P. decursivepinnata*. Numerous reports conform that triploid lily is usually sterile and can be used as a female parent to cross with suitable male parents (Lim et al., 2003; Barba-Gonzalez et al., 2006; Khan et al., 2009b; Xie et al., 2010; Zhou et al., 2011, 2012, 2014; Chung et al., 2013; Xi et al., 2015; Dhiman et al., 2019); Cui et al. (2022) showed that all triploid lilies are partially fertile when used as female parents even they are completely sterile as male parents. The triploid lilies as female parents can be used to cross with appropriate diploid or tetraploid males to produce aneuploid cultivars.

### Performance of Polyploid Hybrids Derived From 2*n* Gametes

Sexual polyploidization has significantly advanced ornamental plant breeding, resulting in a variety of novel cultivars in the market, including those from *Alstroemeria*, *Begonia*, *Chrysanthemum*, *Cymbidium*, *Lilium*, *Phalaenopsis*, *Tulipa*, and others. These cultivars have either unique or larger flowers, different colors, robust growth form, and resistance to different abiotic and biotic stresses. Furthermore, sexual polyploidization has resulted in not only new cultivars, but also new species and new genera, accelerating plant speciation. More than 40 years ago, Roose and Gottlieb (1976) demonstrated that *T. mirus* and *T. miscellus*, two relatively new allotetraploid ornamental plant species had combined allozyme profiles of the diploid parents (*T. dubius* and *T. porrifolius* for *T. mirus*; *T. dubius* and *T. pratensis* for *T. miscellus*). This report documented the link between genotype and biochemical phenotype as well as enzyme additivity. Now, we are in the age of genomics, polyploidization, particularly through the 2*n* gamete route, can cause genomic rearrangement (Soltis et al., 2004), changes in gene content and gene number, alternation in gene expression in combination with actions of transposal elements, small RNAs, and epigenetic regulation (Moghe and Shiu, 2014; Li et al., 2019; Williams, 2021). It is truly believed that such changes and interactions will result in different gene expression profiles, metabolism alternation, and morphology differences. With the art of selection, new cultivars and new plants will be developed. We are not concerned about the evolutionary dead-end of polyploidy, rather we are pursuing a better understanding how polyploidization has rearranged the genetic makeup of plants, how the changes alter gene expression, and subsequently phenotypic variation, and how we can better use 2*n* gametes as a means for developing novel plants and new cultivars.

## Conclusion

The exploitation of 2*n* gametes creates a plethora of opportunities for practical breeding in ornamental plants. Spontaneous production of 2*n* gametes was found in more than 211 accessions belonging to 37genera, 25 families in ornamental plants. The occurrence frequency of 2*n* gametes ranges from 0.03 to 100.00%, depending on genetic and environmental factors. In general, the occurrence percentage of 2*n* gamete in interspecific or intergeneric hybrids was higher than traditional cultivars, but not all of the hybrids produce more 2*n* gametes. Both diploids and polyploids can produce 2*n* gametes, which can be used for producing polyploids with different ploidy levels. Triploids are generally thought to be an evolutionary dead-end, but in practice, they can be used as either the male or female parent in a cross-breeding program to produce sexual polyploids. 2*n* gametes can also be artificially induced by treatment with colchicine, N_2_O, and trifluralin and by manipulation of temperature. Artificial productions of 2*n* gametes are successfully achieved in 61 accessions belonging to 10 genera, nine families with the occurrence frequency ranging from 0.1 to 100%. Triploid, tetraploid, pentaploid, hexaploidy, and octaploid ornamental plants were created by the use of 2*n* gametes. Information gathered from this review shows that polyploid breeding with 2*n* gametes is an efficient and reliable method for ornamental plant breeding. With ongoing research at the molecular level and research toward efficient methods for inducing 2*n* gametes, the importance of 2*n* gametes for ornamental plant breeding will continue to increase in the future.

## Author Contributions

LX, JC, and Z-SZ conceived the idea, edited and refined the manuscript. L-ZK and X-QL conducted literature search and wrote the initial draft. All authors read, corrected, and approved the manuscript.

## Conflict of Interest

The authors declare that the research was conducted in the absence of any commercial or financial relationships that could be construed as a potential conflict of interest.

## Publisher’s Note

All claims expressed in this article are solely those of the authors and do not necessarily represent those of their affiliated organizations, or those of the publisher, the editors and the reviewers. Any product that may be evaluated in this article, or claim that may be made by its manufacturer, is not guaranteed or endorsed by the publisher.
